# Colonial *Microcystis’* biomass affects its shift to diatom aggregates under aeration mixing

**DOI:** 10.1038/s41598-024-53920-5

**Published:** 2024-02-19

**Authors:** Xiaodong Wang, Xuan Che, Jian Zhou, Boqiang Qin, Xiangming Tang, Ziqiu Liu, Xingguo Liu

**Affiliations:** 1 Fishery Machinery and Instrument Research Institute, Chinese Academy of Fishery Sciences, Shanghai, 200092 China; 2 State Key Laboratory of Lake Science and Environment, Nanjing Institute of Geography and Limnology, Chinese Academy of Sciences, Nanjing, 210008 China

**Keywords:** Cyanobacteria blooms, *Microcystis* colonies, Hydrodynamic disturbance, *Nitzschia* aggregates, Nutrients, Low light, Ecology, Environmental sciences, Limnology

## Abstract

The effect of hydrodynamic mixing on controlling *Microcystis* blooms or changing the algal community to diatom dominance has been widely studied; however, the effects of colonial *Microcystis* biomass on the development of the algal community are poorly known. Here, in order to study the changes in *Microcystis* blooms under continuous aeration mixing, an experiment was carried out in a greenhouse with factors of varying biomass of *Microcystis* and inorganic nitrogen and phosphorus enrichment in summer. There were three chlorophyll *a* (Chl-*a*) levels in six treatments: low Chl-*a* level of 68.4 μg L^-1^ (treatments L, L-E), medium Chl-*a* level of 468.7 μg L^-1^ (treatments M, M-E), and high Chl-*a* level of 924.1 μg L^-1^ (treatments H, H-E). Treatments L-E, M-E and H-E were enriched with the same inorganic nitrogen and phosphorus nutrients. During the experiment of 30 days, the concentration of *Microcystis* and Chl-*a* decreased, and diatom *Nitzschia palea* cells appeared in all the treatments, which became dominant in treatments M, M-E, H and H-E, with the highest biomass of 9.41 ± 1.96 mg L^-1^ *Nitzschia* in treatment H-E on day 30. The rank order of the biomass of *Nitzschia* from low to high was (L = L-E) < (M = M-E) < H < H-E (*P* < 0.05). In addition, *Nitzschia* cells were aggregates attached to *Microcystis* colonies in all the treatments. The results showed that the initial biomass of colonial *Microcystis* affected the algal shift from *Microcystis* dominance to *Nitzschia* dominance. However, the enriched inorganic nitrogen and phosphorus was beneficial for the *Nitzschia* increase in the high biomass treatment alone. The shift from *Microcystis* dominance to diatom dominance under continuous aeration mixing may be caused by low light conditions as well as the nutrients released from *Microcystis* decay. Moreover, the aerobic condition caused by aeration mixing maintained the colonial mucilaginous sheath to support the growth of *Nitzschia* cells in aggregation. This study found for the first time that *Microcystis* blooms could shift to diatom *Nitzschia* dominance in aggregates. It provided a method to control and manipulate *Microcystis* blooms to diatom dominance through continuous aeration mixing to proper biomass of *Microcystis* colonies. The shift to diatoms dominance would provide more high quality food organisms for aquaculture and be beneficial to the material cycling and energy flowing in food web dynamics.

## Introduction

Cyanobacterial blooms of *Microcystis* are a widespread global phenomena and contributor to various environmental problems^1–5^. It is well-documented that *Microcystis* can impact a wide range of aquatic taxa including zooplankton and fish species, and humans due to the toxic chemical it can produce called microcystins^6–8^. Microcystins continue to be a worldwide concern because it can cause a range of sublethal to lethal effects in organisms, and it remains unknown what impacts these toxins can have in wild populations^9,10^. Consequently, a variety of approaches have been taken—including physical, chemical, biological, or in combination of measures—to regulate or control cyanobacterial blooms from deteriorating ecosystem health^11–13^.

Hydrodynamic alterations has been suggested to play a key role in regulating algal communities. The hydrologic regimes were shown to be the main driving force in the succession of the phytoplankton community in the Three Gorges Reservoir (TGR) of China^14^. Water flow regimes can reshape the phytoplankton community compositions^15^. Moreover, the hydrodynamic disturbance may be a key ecological approach to control *Microcystis* blooms or dominance, which enable non-cyanobacteria phytoplankton, diatoms or diatoms and green algae to outcompete cyanobacteria^16–20^.

The obvious feature of *Microcystis* bloom is that it consists of fine particles of *Microcystis* colonies, which can be up to several millimeters and float on the water instead of being suspended in the water column if the hydrodynamic force is not strong enough. And the algal shift from *Microcystis* dominance to diatoms or diatoms and green algae dominance was related to such reasons: (a) *Microcystis* colonies lose their advantage of buoyancy of floating on the water under artificial turbulence^21^; (b) The negatively buoyant algae, such as green algae and diatoms, profited from the mixed conditions with fluctuating irradiance^18,21^. Visser et al.^22^ reviewed the control of cyanobacteria blooms by artificial mixing, including the algal shift mechanisms.

However, the opinion that artificial mixing was an effective solution to control harmful *Microcystis* blooms was not unanimous, and that the hydrodynamic disturbance influencing blooms has not been fully understood^23^. Hydrodynamic disturbance may have a completely different impact on the *Microcystis* dominance, which may improve *Microcystis* dominance formation. For example, the tropical cyclones stimulated *Microcystis* blooms in hypertrophic Lake Taihu, China^24^. Moreover, the disturbance frequency and intensity on the algal shift process may have varying effects^25,26^, in which continuous hydrodynamic mixing weakened the dominance of *Microcystis*^26^ and intermittent disturbance benefited colony size, biomass, and dominance of *Microcystis* in Lake Taihu under simulated field conditions^25^.

Meanwhile, the abundance of *Microcystis* can be an important factor affecting the algal shift under hydrodynamic disturbance^21^. Our initial study showed that the biomass of bloom-forming colonial *Microcystis* affected its response to aeration disturbance, in which the biomass of diatom *Nitzschia* was the highest when the initial chlorophyll *a* (Chl-*a*) of the bloomed *Microcystis* was 346.8 μg L^-1^, in comparison with those of 32.5, 1413.7, and 14,250.0 μg L^-1^, respectively^27^. Then the interval between 346.8 and 1413.7 μg L^-1^ was so large, what would happen to the *Microcystis* colonies if the biomass level was between 346.8 and 1413.7 μg L^-1^? There may be more diatom *Nitzschia* shift from the *Microcystis* colonies if the *Microcystis* colonies’ biomass level was between 346.8 and 1413.7 μg L^-1^ in Chl-*a*. Then we hypothesized that the initial *Microcystis* biomass can affect the algal shift under aeration mixing, and there was a more suitable *Microcystis* biomass level to achieve the shift from *Microcystis* dominance to diatom dominance.

In the review of Visser et al.^22^, the effect of the biomass of colonial *Microcystis* on the algal shift was not included, although the effects of artificial mixing on algal biomass changes were reviewed. The algal succession in *Microcystis* blooms or dominance of different biomass under hydrodynamic disturbance was not fully understood, as many factors affect the shift process, such as nutrients, buoyancy regulation, temperature, oxygen, light, and so on^22^. Moreover, biomass can change the nutrients level, which can affect the algal community composition^28–30^.

In order to obtain a more suitable range of *Microcystis* biomass that can promote more diatom succession and clarify if inorganic nitrogen and phosphorus nutrients enrichment can improve the algal shift, an experiment with varying biomass of colonial *Microcystis*, particularly the Chl-*a* level between 346.8 and 1413.7 μg L^-1^, coupling inorganic nitrogen and phosphorus enrichment, was carried out in a greenhouse, which can provide high temperature to improve the algal shift process. Then the mechanism of the algal shift from *Microcystis* dominance to other algae dominance would be clearer.

Moreover, diatoms, different from *Microcystis* containing microcystins, can produce high-value compounds, including chrysolaminarin (Chrl), eicosapentaenoic acid (EPA), and fucoxanthin (Fx), which can be applied in aquaculture, human health foods, pharmaceuticals, and even cosmetics^31^. In addition, *Nitzschia* are common food organisms in aquaculture^32^. Therefore, if the shift from *Microcystis* dominance to *Nitzschia* dominance was achieved, the diatoms would provide more high quality food organisms for aquaculture, which is also beneficial to the material cycling and energy flowing in food web dynamics in algal blooming waters.

## Materials and methods

### Experimental design

On the 8th of August, 2019, a thick *Microcystis* bloom was obtained from an aquaculture pond breeding mainly *Megalobrama amblycephala*, located in the Songjiang District of Shanghai, China. The bloom was dominated by *Microcystis* spp., particularly *M. aeruginosa*, and *Microcystis* made up 99.0% of the total biomass, while *Anabaena* accounted for 1.0%. On the 10th of August, 2019, the thickly bloomed *Microcystis* was transferred to transparent borosilicate 10 L glass jars (23 cm diameter, 35 cm high) in a greenhouse of 220 m^2^. A layer of plastic film was affixed on the glass of the greenhouse to block the sunlight. The experiment ended on the 9th September, 2019, which lasted 30 days. Tap water was used to dilute the thick bloom to obtain different biomass of *Microcystis*. The concentrations of total nitrogen (TN) and total phosphorus (TP) in the tap water were 1.088 and 0.011 mg L^-1^, respectively.

Chlorophyll-*a* (Chl-*a*) concentration was chosen to reflect the biomass of the *Microcystis* bloom. There were three chlorophyll *a* (Chl-*a*) levels in six treatments (Table 1): low Chl-*a* level of 68.4 μg L^-1^ (L, L-E), medium Chl-*a* level of 468.7 μg L^-1^ (M, M-E), and high Chl-*a* level of 924.1 μg L^-1^ (H, H-E). Treatments L-E, M-E and H-E were enriched with the same inorganic nitrogen and phosphorus nutrients. Each treatment has three replicates. The initial TN, TP, and Chl-*a* concentration of the treatments is shown in Table 1.

**Table 1:** The concentration of Chl-*a*, TN and TP in each treatment at the start of the experiment, showing whether the treatment was nutrient enriched (mean ± SD).

Treatment	Chl-*a* (μg L^-1^)	TN(mg L^-1^)	TP(mg L^-1^)	Nutrients enrichment
L	68.37 ± 1.97	2.952 ± 0.093	0.200 ± 0.051	No
L-E	68.37 ± 1.97	2.952 ± 0.093	0.200 ± 0.051	Yes
M	468.67 ± 27.62	14.382 ± 0.191	0.982 ± 0.075	No
M-E	468.67 ± 27.62	14.382 ± 0.191	0.982 ± 0.075	Yes
H	924.09 ± 11.52	28.360 ± 0.698	1.990 ± 0.171	No
H-E	924.09 ± 11.52	28.360 ± 0.698	1.990 ± 0.171	Yes

During the experiment, treatments L-E, M-E and H-E were enriched with 2.1 mg/L nitrogen (N) and 0.3 mg/L phosphorus (P) every three days from 15th August on, which was in the form of NH_4_Cl and KH_2_PO_4_, respectively. Nine times of nutrients were enriched, with a total of 189 mg N and 27 mg P to satisfy the growth of algae. Thus, the concentration of TN and TP in treatment L-E was next to that in treatment M, and that in treatment M-E was close to treatment H.

Each jar was continuously aerated with a bubble stone. The aeration intensity was approximately 0.35 m^3^ h^-1^ all the time, to maintain the suspension of *Microcystis* colonies as much as possible and prevent water splashing. No sediment was provided. Ultrapure water was used to replenish that lost by evaporation about every 3 days.

### Shading rate in the greenhouse and its measurement.

The light intensity in the greenhouse was real-time changing, and the photosynthetically available radiation (PAR) were measured both inside the greenhouse and outdoors at about 11:00 h and 13:30 h, on a sunny day, the 14th of August, 2019, to show the shading rate in the greenhouse. PAR quantum (unit: μMol m^-2^ s^-1^) and PAR energy (unit: W m^-2^) was measured by a Spectrosense2 meter associated with a four-channel sensor (Skye Instruments, UK). It was measured three times in three minutes, and the average values were calculated to obtain the light transmittance, which was the ratio of the light intensity in the greenhouse to outdoors. Then shading rate was calculated as (%): shading rate (%) = 100 − transmittance (%).

### Water quality and Chl-a measurements

During the experiment, water temperature (WT), dissolved oxygen (DO), salinity (Sal) and pH were measured at about 14:00 h every 2 days with a YSI multi-parameter water quality monitor meter (YSI professional plus, Yellow Spring Instruments, USA) in situ. The nutrient variables such as total nitrogen (TN), total phosphorus (TP), dissolved TN (DTN), dissolved TP (DTP), ammonia nitrogen (NH_4_^+^-N), and soluble reactive phosphorus (SRP) were measured every 6 days. Water for analysis of DTN, DTP, NH_4_^+^-N and SRP was filtered through 0.45 μm mixed fiber filters, which were washed with deionized water before using. Measurements of TN, TP, DTN and DTP were taken following the methods of Gross and Boyd^33^, while NH_4_^+^-N was determined by Nessler's reagent spectrophotometry and SRP was determined by molybdenum–antimony–ascorbic acid colorimetry^34^. A PHYTO-PAM (Waltz, Effeltrich, Germany) chlorophyll fluorescence meter with the software phytowin2.13 was used to measure Chl-*a*.

### Algae identification and counting

Algal samples were collected six times during the experiment, with a sampling frequency of every 6 days. The algal samples at the start and on day 6 were mixed samples of the aliquots from 3 jars, as well as those in treatments L and L-E on day 12, and the other samples were from the jars in 3 replicates.

To determine algal density, 50 mL water samples were preserved with 1% Lugol’s solution and stored in darkness until analysis. For enumeration, two replicate aliquots were enclosed in 0.1 mL plankton counting chambers that were modified from the Palmer and Maloney design^35^. Most cells were observed at 400 × magnification by an optical microscope Olympus CX31 (Olympus, Japan), while large algal cells or colonies were observed at 100 × magnification. And some microphotographs were taken under 400 × magnification. The cells were mainly identified to the genus level as referenced by morphologies^36^. For the enumeration of cells in *Microcystis* colonies, subsamples were heated to 60 °C for 2–4 h to disintegrate the colonies.

Algal volumes were calculated based on cell density and cell size measurements. Calculation of cell volumes was according to their shape, and measurements of length, height, and diameter were obtained to calculate the volume. At least 30 algal units were measured to obtain the average cell volume for each genus or species. The conversion to wet weight biomass assumed that 1 mm^3^ of volume was equivalent to 1 mg of wet weight biomass^29^.

### Data analysis

Data comparison among the treatments was conducted with SPSS 24.0 software for Windows (Statistical Product and Service Solutions, IBM, New York, USA) using two-factor analysis of variance (ANOVA) (biomass × time) in the general linear model. To improve the homogeneity of variances, most data of each treatment were square root transformed before the comparison, and the cell density ratio of *Microcystis* to *Nitzschia* was log10 transformed, while the biomass ratio of *Microcystis* to *Nitzschia* was fourth root transformed^37^. The data were mainly shown as mean ± SD, except some algal wet weight was shown as mean. The comparison of the algal wet weight was for the data from days 12 to 30. The Least-significant difference (LSD) test was chosen for pairwise comparisons.

## Results

### Light in the greenhouse

The glass and the affixed plastic film on the glass of the greenhouse shaded the sunshine, and the shading rate of the greenhouse was about 72 ~ 74% (Table 2).

**Table 2:** The PAR both in the greenhouse and the outdoor, and the calculated shading rate of the greenhouse on August 14, 2019.

Time	PAR	In the greenhouse	Outdoors	Shading rate of the greenhouse (%)
11:00	Quantum/(μmol·m^-2^·s^-1^)	473.03 ± 7.84	1744.30 ± 6.18	72.9
	Energy/(W·m^-2^)	82.99 ± 1.63	328.78 ± 0.75	74.8
13:30	Quantum/(μmol·m^-2^·s^-1^)	466.03 ± 5.70	1670.90 ± 17.46	72.1
	Energy/(W·m^-2^)	80.93 ± 2.20	312.35 ± 3.15	74.1

### Water quality

**Figure 1:**
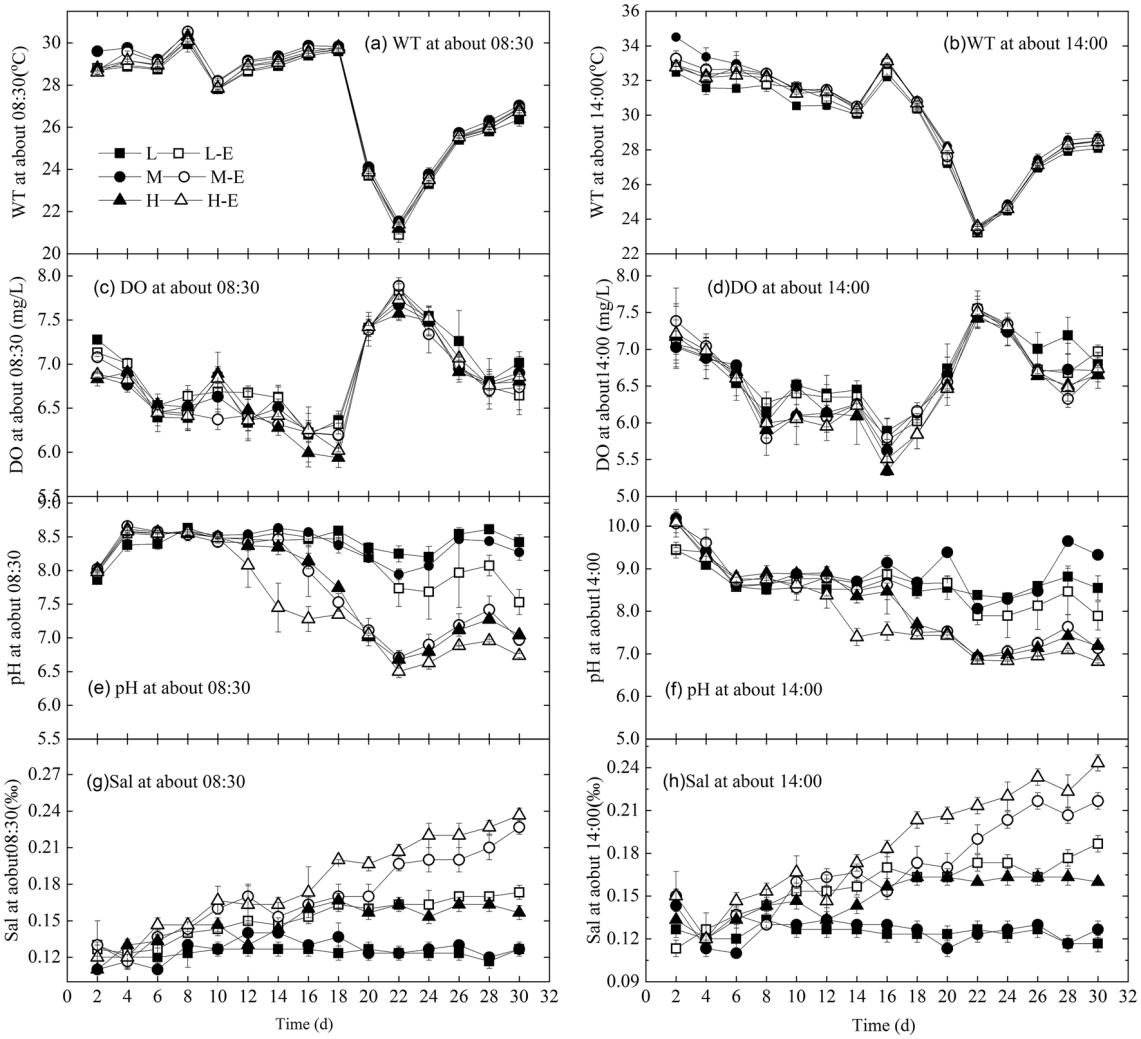
The changes in WT, DO, Sal, and pH values at 8:30 a.m. and 14:00 p.m. of each treatment.

The WT in the greenhouse was real-time changing, and the range of WT was 20.5 ~ 34.6 °C during the experiment (Fig. 1a,b), and the WT at 14:00 was 2 ~ 5 °C higher than that at 08:30. DO ranged in 5.29 ~ 7.98 mg L^-1^ (Fig. 1c,d), showing that the *Microcystis* bloom was in aerobic instead of anaerobic condition.

The pH values were in the range of 5.73 ~ 10.31 (Fig. 1e,f). The rank order from high to low in pH values was (L, M) > L-E > (M-E, H) > H-E (*P* < 0.05) in the morning, while that was M > L > L-E > (M-E, H) > H-E (*P* < 0.05) in the afternoon, showing the pH values decreased with the *Microcystis* biomass increase and the inorganic nutrient enrichment.

Sal ranged from 0.11 to 0.26‰ (Fig. 1g,h), and the rank order of Sal from high to low was H-E > M-E > L-E > H > (L, M) (*P* < 0.05) both in the morning and the afternoon, showing the *Microcystis* biomass increase and inorganic nutrient enrichment improved the Sal significantly (*P* < 0.05).

**Figure 2:**
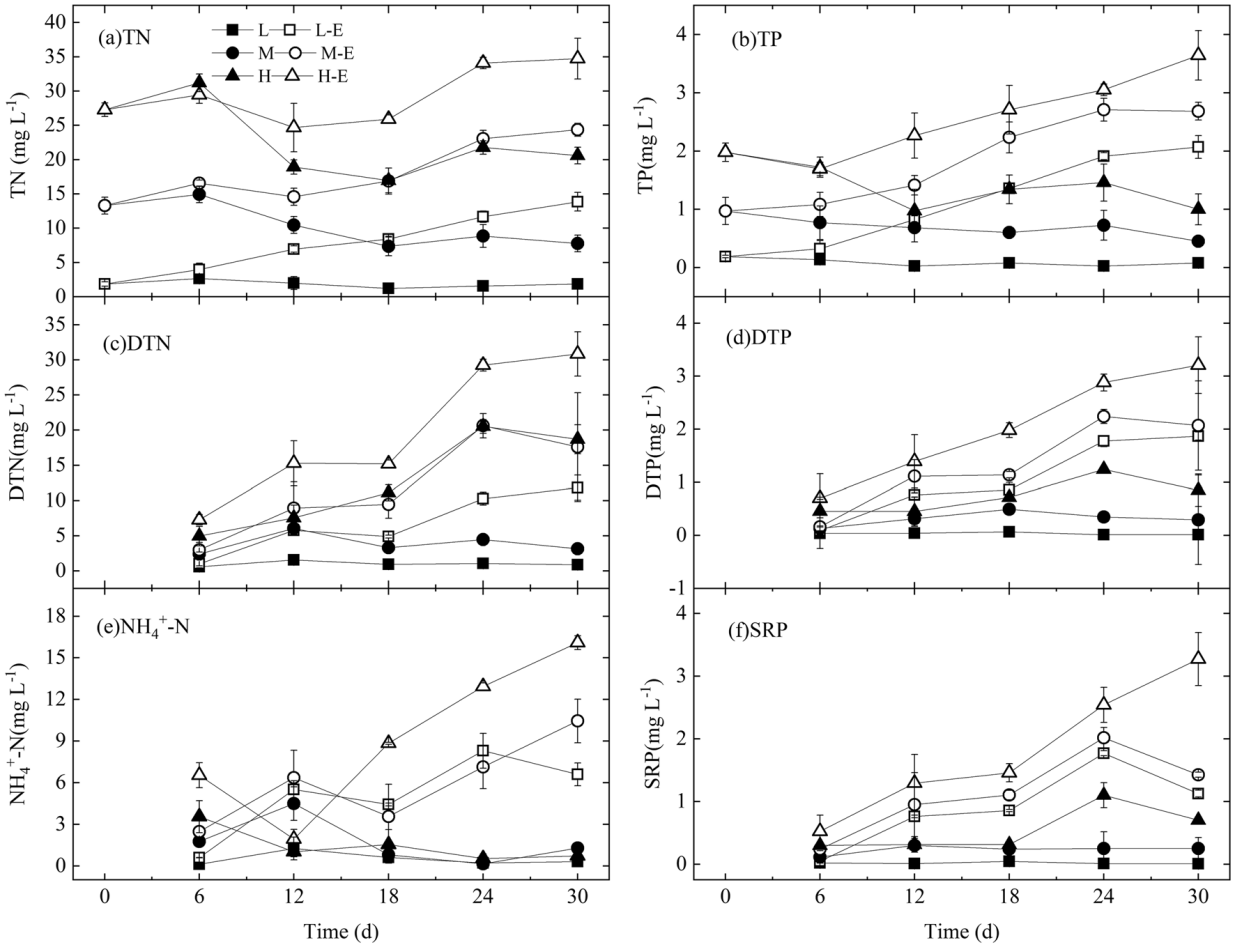
The changes in TN, TP, DTN, DTP, NH_4_^+^-N, and SRP of each treatment.

During the experiment, the TN concentration in treatments L, L-E, M, M-E, H and H-E was 1.850 ± 0.834, 10.341 ± 4.659, 9.686 ± 2.943, 20.652 ± 5.475, 21.863 ± 4.986 and 30.937 ± 5.063 mg L^-1^, respectively; and TP was 0.081 ± 0.056, 1.533 ± 0.830, 0.723 ± 0.258, 2.269 ± 0.917, 1.336 ± 0.398 and 3.193 ± 1.446 mg L^-1^, respectively (Fig. 2). The nutrients level in treatment L, M and H of no nutrients enrichment was relatively stable (Fig. 2). In the later stage, the TP, DTP, NH_4_^+^-N and SRP in treatment L-E were significantly higher than treatment H (*P* < 0.05), and the TN and DTN concentration in treatment L-E were between treatment M and H (*P* < 0.05).

The comparison result showed the rank order from high to low in TN was H-E > (M-E, H) > (L-E, M) > L (*P* < 0.05), showing the N enrichment in treatment L-E and M-E increased the TN to the level in treatment M and H, respectively. The rank order for TP was H-E > M-E > (H, L-E) > M > L (*P* < 0.05), showing the P enrichment in treatment L-E improved the TP to the level in treatment H.

### Chl-a

**Figure 3:**
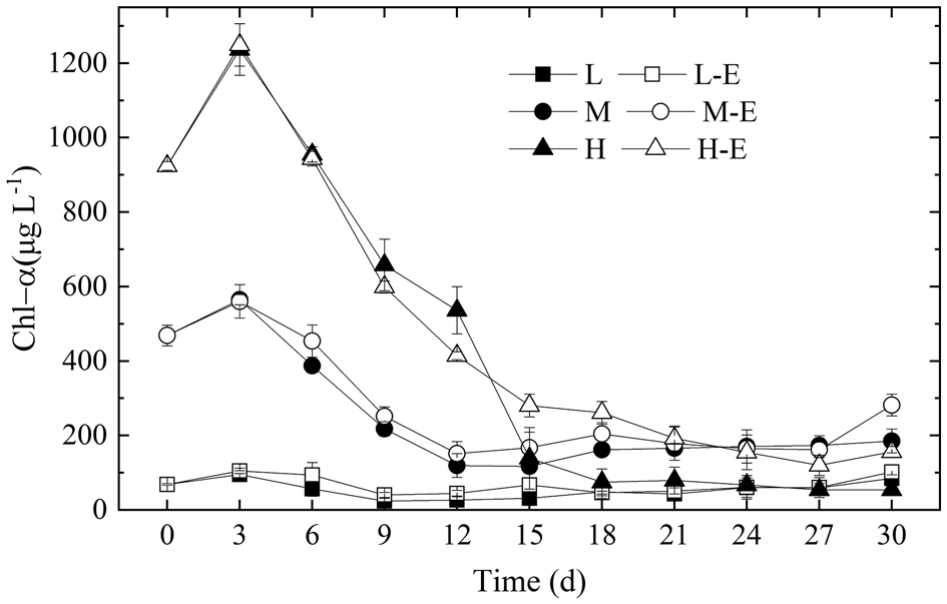
Changes in Chl-*a* of each treatment during the experiment.

The Chl-*a* concentration in all the treatments gradually decreased until on about day 15, and remained relatively stable from days 18 to 30 (Fig. 3). The analysis result on Chl-*a* from the start to day 15 showed that Chl-*a* in treatment L was significantly lower than the other 5 treatments (*P* < 0.05). The Chl-*a* in treatments H and H-E was significantly higher than both treatments M and M-E (*P* < 0.001), and that in treatments M and M-E was significantly higher than both treatments L and L-E (*P* < 0.001). These showed the *Microcystis* biomass was the main factor affecting the Chl-*a* content. Then from days 18 to 30, there was no significant difference between treatments L, L-E and H in Chl-*a* content (*P* > 0.05), while they were all significantly lower than that in treatments M, M-E and H-E (*P* < 0.001).

### Algal succession

At the beginning of the experiment, there were very few cells of *Planktothrix*, *Chlamydomonas* and *Pseudanabaena* besides the dominance of *Microcystis*. During the experiment, the phytoplankton was dominantly composed of Cyanobacteria, Chlorophyta, and Bacillariophyta, of which Cyanophyta and Bacillariophyta were the most dominant ones. The Cyanophyta were primarily composed of *Microcystis,* while the Bacillariophyta were mainly *Nitzschia*. There were a few green algae during the experiment, particularly in the low biomass treatments L and L-E, which were not counted.

**Figure 4:**
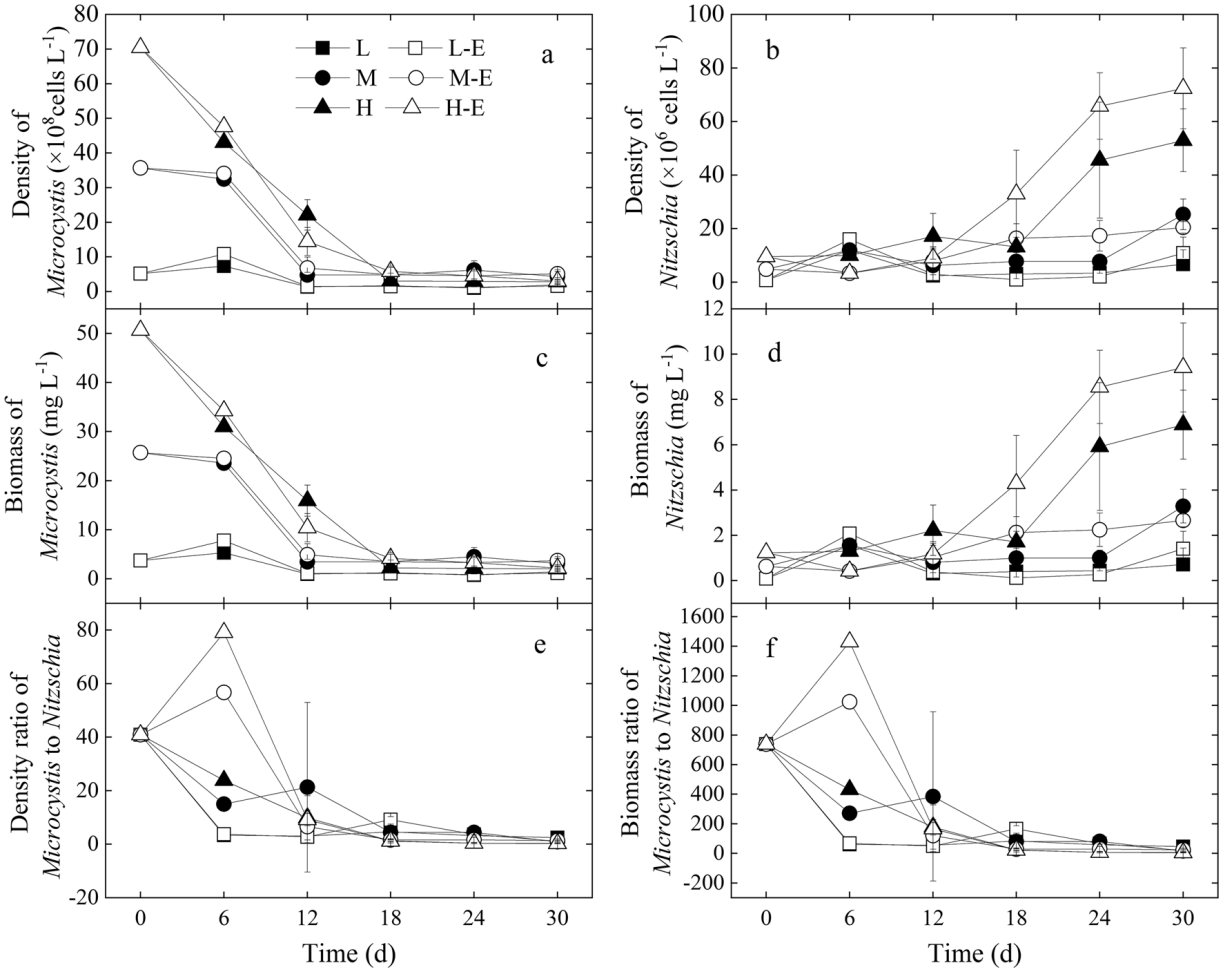
Changes in density of *Microcystis* (**a**) and *Nitzschia* (**b**), biomass of *Microcystis* (**c**) and *Nitzschia* (**d**), density ratio of *Microcystis* to *Nitzschia* (**e**), and the biomass ratio of *Microcystis* to *Nitzschia* (**f**) in each treatment during the experiment.

In this experiment, the *Microcystis* colonies gradually decomposed, and the genus *Nitzschia*, primarily *Nitzschia palea* grew in the *Microcystis* colonies. The changes in the cell density and wet weight of the two genera *Microcystis* and *Nitzschia* showed that the biomass of *Microcystis* gradually decreased while that of *Nitzschia* gradually increased (Fig. 4). And *Nitzschia* dominated in all the treatments from day 18 on (Fig. 4).

The analysis results showed the wet weight of *Microcystis* in treatments L and L-E was the lowest (*P* < 0.05), and that in treatments H and H-E was the highest (*P* < 0.05), showing the change in wet weight of *Microcystis* under aeration mixing depended on the initial *Microcystis* biomass. And the nutrients’ enrichment in treatments H-E, M-E and L-E did not decrease the biomass of *Microcystis*, in comparison to treatments H, M and L, respectively (*P* > 0.05).

Basically, the higher the initial biomass of *Microcystis* in treatments L, M and H, the higher the biomass of *Nitzschia* in the later stage of the experiment (*P* < 0.05). Moreover, the biomass of *Nitzschia* in treatment H-E was significantly higher than treatment H (*P* < 0.05), while both of them were significantly higher than treatments M and M-E (*P* < 0.001). These showed the N and P enrichment was beneficial for the *Nitzschia* increase in the high biomass treatment H (*P* < 0.05). However, the nutrient enrichment in treatment M-E did not increase the *Nitzschia* biomass in comparison to treatment M, nor did it in treatments L-E to L (*P* > 0.05).

Both the cells’ density ratio and biomass ratio of *Microcystis* to *Nitzschia* gradually decreased to be close to each other in all the treatments (Fig. 4). The density ratio of *Microcystis* to *Nitzschia* in treatment H-E can be near 1.0, and the biomass ratio of *Microcystis* to *Nitzschia* in treatment H-E can be lower than 1.0 (Fig. 4). The comparison result in density ratio and biomass ratio of *Microcystis* to *Nitzschia* showed the values in treatments H and H-E were the lowest (*P* < 0.05), and they were all significantly lower than the other four treatments (*P* < 0.05). The values in treatments L, L-E, M, and M-E were not significantly different from each other (*P* > 0.05).

**Figure 5:**
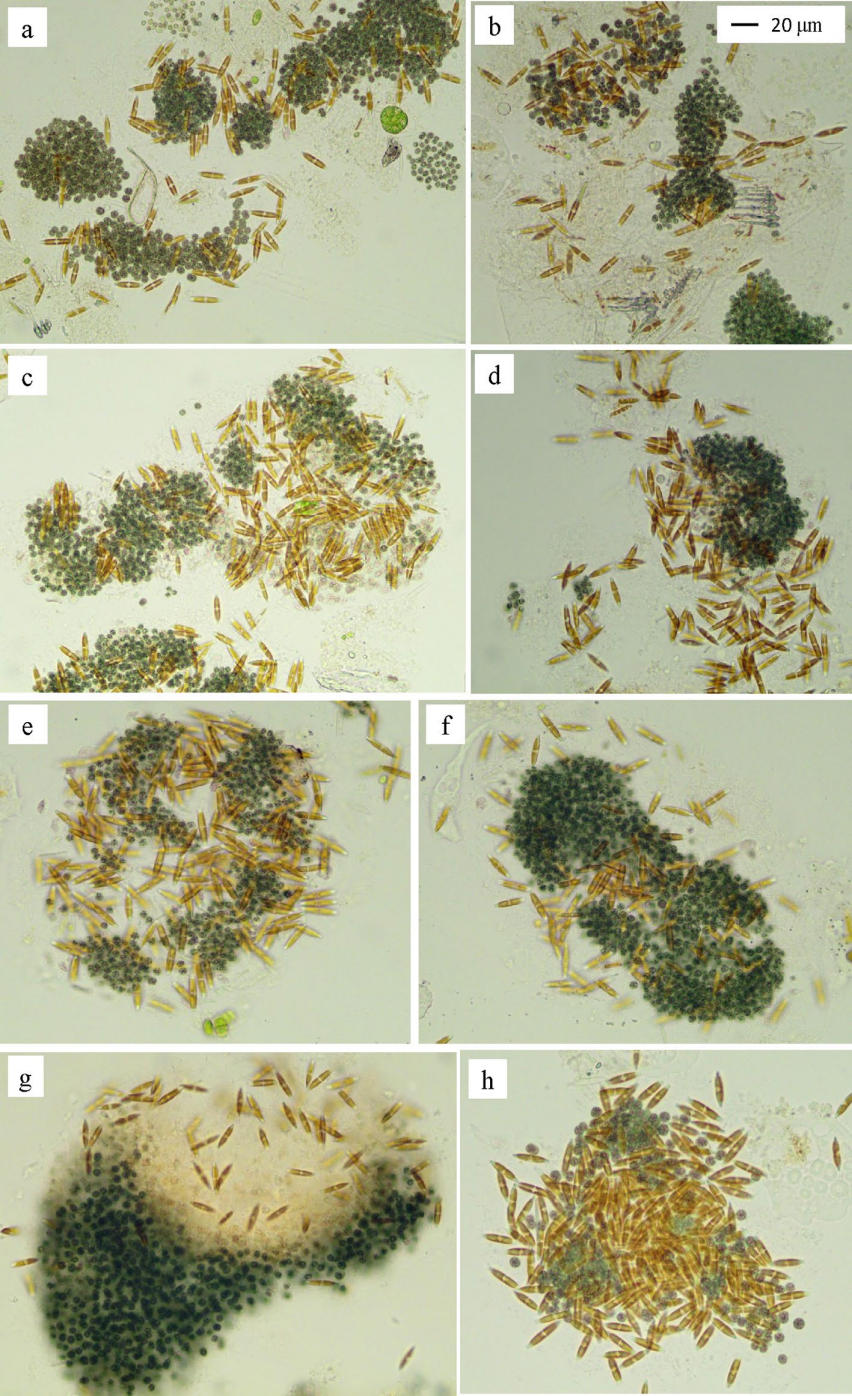
Some representative pictures on the aggregates of *Nitzschia* cells in *Microcystis* colonies in treatment H (**a**–**d**) and treatment H-E (**e**–**h**) on day 20.

**Figure 6:**
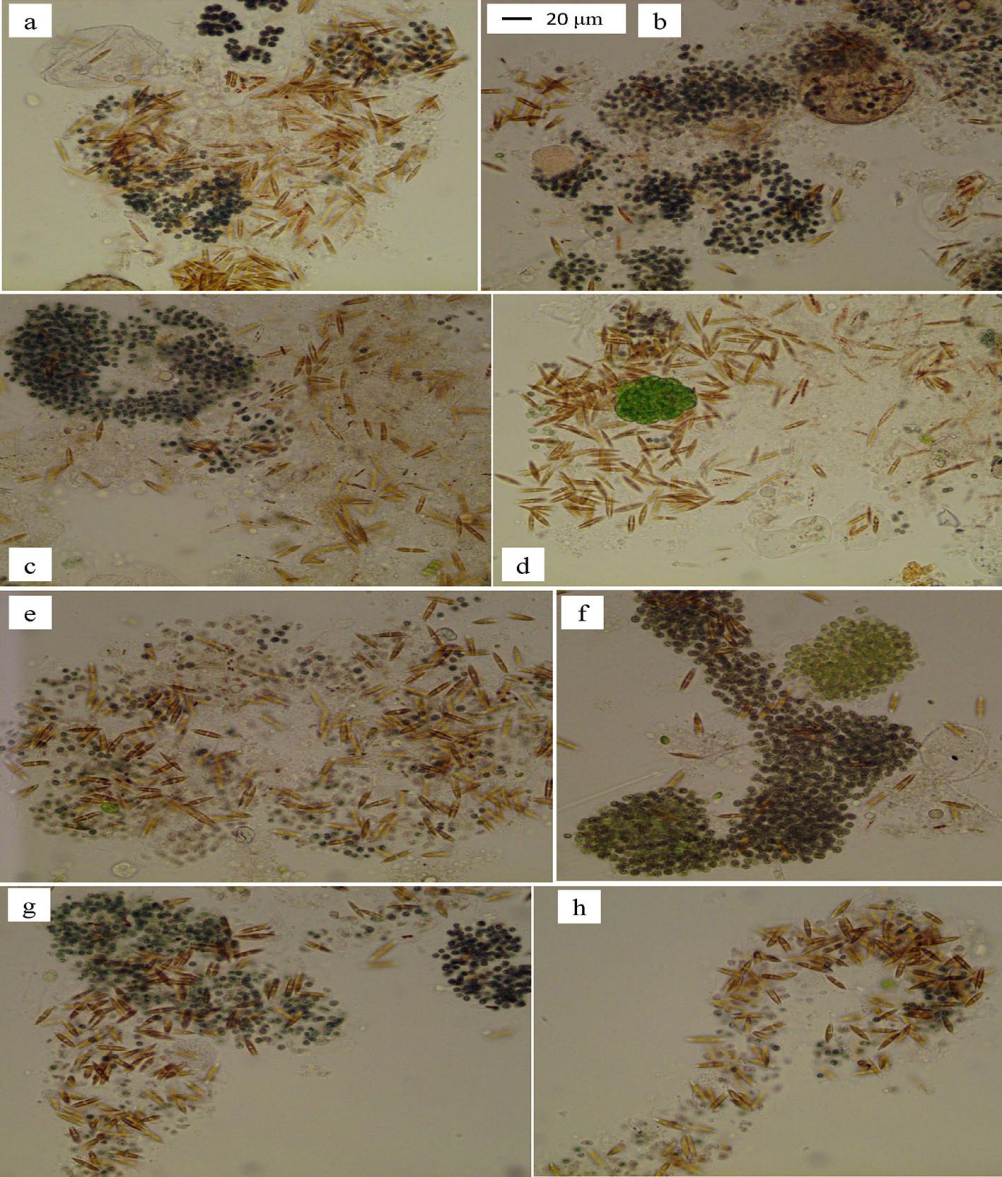
Some representative pictures on the aggregates of *Nitzschia* cells in *Microcystis* colonies in treatment H (**a**–**d**) and treatment H-E (**e**–**h**) on day 24.

Moreover, the *Nitzschia* cells were not in free-living forms but attached to the *Microcystis* colonies in almost all the treatments. The aggregates of *Nitzschia* cells in the *Microcystis* colonies in treatments H and H-E are shown in Figs. 5 and 6.

## Discussion

In this experiment, the colonial *Microcystis* shift to diatom dominance in all treatments with aeration mixing in the greenhouse (Figs. 4, 5, 6); moreover, the initial colonial *Microcystis* biomass affected the *Nitzschia* biomass (Fig. 4). This was similar to our earlier results in Wang et al.^27^, which showed that the biomass of bloom-forming colonial *Microcystis* affected its response to aeration disturbance, and diatom *Nitzschia* appeared in the *Microcystis* colonies when the initial Chl-*a* of the bloomed *Microcystis* was 346.8 μg L^-1^. The *Nitzschia* biomass in treatments M, M-E, H, H-E was much higher than that in Wang et al.^27^, showing the initial colonial *Microcystis* biomass in treatments M, M-E, H, H-E was more suitable to promote the diatom succession. Thus, in comparison with the results in Wang et al.^27^, more diatom *Nitzschia* shift from the *Microcystis* colonies when the *Microcystis* colonies’ biomass was 468.7 and 924.1 μg L^-1^ in Chl-*a*. And it can be inferred that the *Microcystis* biomass level between 468.7 and 924.1 μg L^-1^ in Chl-*a* was also suitable to achieve the shift from *Microcystis* dominance to *Nitzschia* dominance.

The fact that the initial *Microcystis* biomass affects the diatom succession and *Nitzschia* cells adhere to *Microcystis* colonies is very interesting. Similarly, the abundance of *Microcystis* affected the algal shift under hydrodynamic disturbance at Hartbeespoort Dam in South Africa^21^. When *Microcystis* abundances were high, the diatoms *Cyclotella meneghiniana* and *Melosira* (syn. *Aulacoseira*) *granulata* and the cryptophytes *Chroomonas* sp. and *Cryptomonas* sp. occurred more frequently^21^. On the contrary, when *Microcystis* was rare, some green algae tended to increase across a broad spectrum of temperature and nutrient conditions^21^.

Moreover, many studies have found that hydrodynamic mixing caused the algal shift from cyanobacteria, particularly *Microcystis* bloom, to diatom or/and green algae dominance^16–18,22,38^. Mixing shifts the *Microcystis* blooms from Lake Taihu, China, to the dominance of diatoms and green algae^20^. Diatoms such as *Asterionella*, *Fragilaria*, and *Staurastrum* were favored by deep mixing, which hampered the cyanobacteria (*Microcystis* and *Anabaena*)^39^. The growth of *Microcystis* in Nieuwe Meer Lake of the Netherlands was reduced by artificial disturbance^17^, and high-intensity hydrodynamic disturbance changed the *Microcystis* bloom to a diatom and green algae bloom^16,18^. The cyanobacteria bloom in Ford Lake shifts to a diatom bloom with artificial disturbance^19^. Continuous hydrodynamic mixing weakened the dominance of *Microcystis*, which was beneficial for the other algae^26^. Artificial aeration replaced the dominant bloom-forming cyanobacteria with diatoms in a small tropical reservoir^40^.

However, the roles of cyanobacteria biomass on the shift from cyanobacteria dominance to non-cyanobacteria dominance under artificial mixing were not considered by Visser et al.^22^, and how the algae reacted to turbulence mixing is not fully understood^23,41^, although that turbulence mixing plays a vital role in influencing the algal growth rate is widely accepted. The reasons for hydrodynamic turbulence favoring the dominance of diatoms and green algae other than buoyant cyanobacteria were summarized by some researchers^20,22^, which include buoyancy regulation, competition for nutrients, competition for light, sedimentation losses, and so on. In this experiment, aeration mixing directly caused the aggregates of *Microcystis* colonies to suspend and keep rolling in the water. And the indirect impact includes the influence from changes in nutrient level, suspended particulate matter, light shading, DO, and so on.

Many factors affected the algal shift from *Microcystis* dominance to diatom dominance under aeration mixing, and nutrients are one of the important factors. The decrease of Chl-*a* and increase of NH_4_^+^-N and SRP in all the treatments expressed the decay of the *Microcystis* colonies. Moreover, the higher the *Microcystis* biomass, the more dissolved nutrients release (Fig. 2). These dissolved nutrients were the material basis for the algal shift. Similarly, algal decomposition can release some nutrients to support other algae^24^. In treatments L, M, and H with no inorganic nutrients enrichment, the released nutrients from *Microcystis* supported the algal shift to *Nitzschia* dominance (Figs. 2 and 3). The inorganic nutrient enrichment in treatment H-E improved the biomass of *Nitzschia* in comparison with treatment H; on the other hand, the enrichment in treatment M-E did not improve the biomass of *Nitzschia* in comparison with treatment M (Fig. 4). These indicated that it's not always the case that the higher the concentration of inorganic nitrogen and phosphorus nutrients, the better for the diatom succession.

Light is also an important factor affecting the algal shift. In this experiment, the aggregates of *Microcystis* colonies can be physically mixed into the water column with the aeration mixing, which produces “self-shading”. And it is sure that the higher the *Microcystis* biomass, the lower the light intensity in the jars. The biomass of colonial *Microcystis* affected the light intensity in the jars as well as the nutrients’ level. Moreover, the greenhouse was in low light with a shading rate of about 72~74% in comparison with the light outdoors. It’s probable that the low light satisfied the light demand for the diatom growth, as diatoms and green algae are considered to be better adapted to fluctuating light conditions in comparison to buoyant cyanobacteria^18^. Diatoms are also considered to prefer to low light conditions, and high irradiance contributed to the decline of the spring diatom^42^. Huisman et al.^18^ established a light competition theory, which meant that light instead of nutrients would be the limiting factor for algae growth in highly eutrophic conditions with full mixing. Mixing affected the light competition between buoyant and sinking phytoplankton species in eutrophic waters^18,43^.

Moreover, the algal response to aeration mixing depended on the algal group’s characteristics. Compared to other algal groups, cyanobacteria were regarded as relatively more sensitive to hydrodynamic turbulence^44^. Some information for the development of phytoplankton communities in turbulent water was provided by Kang et al.^41^, which studied the reconstruction of phytoplankton under the dual effects of turbulence and suspended particulate matter, and found that the turbulent treatments promoted the shift from *Microcystis* sp. dominance to *Scenedesmus* sp. and *Chlorella* sp. dominance.

The shift from *Microcystis* dominance to diatom dominance in this experiment is very distinctive, in which the *N. palea* cells formed aggregates in the mucilaginous sheath of *Microcystis* colonies instead of free-living (Figs. 5 and 6). The aggregation of cells in *Microcystis* colonies is due to the mucilaginous sheath, which mainly consists of EPS (extracellular polymeric substances)^45,46^. In comparison to the decomposition of a high biomass of *Microcystis* colonies in anoxic or anaerobic conditions^47^, the decomposition under aerobic conditions was much slower^27^. The aerobic conditions caused by the aeration mixing slowed down the decomposition of *Microcystis* colonies in comparison with anoxic or anaerobic conditions, in which the EPS of *Microcystis* colonies provided a physical medium for the diatoms’ aggregation under aeration mixing (Figs. 5 and 6).

Similarly, diatoms coexisted with *Microcystis* in African freshwater lakes, as well as the filamentous cyanobacterium *Pseudoanabaena* sp. coexisted with *Microcystis* colonies by adhesion way^48^. Diatom *Nitzschia* is a common genus that forms aggregates in marine snow^49^, and it’s considered that the diatoms are accompanied by the production of a large number of EPS. Transparent exopolymer particles (TEP) are a large class of EPS with high stickiness that promotes the formation of diatoms aggregates in marine snow^49,50^. However, the EPS form the diatom aggregation in this experiment comes from the *Microcystis* colonies, which was not released by the diatoms. This experiment not only provides an appropriate biomass of *Microcystis* bloom for the shift from *Microcystis* dominance to diatom dominance, but also shows that the EPS of the *Microcystis* colonies provides aggregating matrix for the diatoms, which is similar to the formation of marine snow with diatom aggregation.

Moreover, the phenomenon of diatoms adhering to algal blooms also occurs in the marine red tide species, *Phaeocystis*. In marine red tide research, diatoms utilize *Phaeocystis* colonies not only as habitat, but that they were able to utilize the colonial matrix as a growth substrate^51^. Numerous studies have found that diatoms, including *Nitzschia* can attach to and grow on *Phaeocystis* colonies^52,53^, and the mechanism may be similar to the *Nitzschia* aggregates in this experiment.

## Conclusions

This study showed that the algal shift in colonial *Microcystis* blooms under aeration mixing was affected by the initial biomass, and when the initial *Microcystis* biomass was appropriate, such as at Chl-*a* levels of 468.7 μg L^-1^ and 924.1 μg L^-1^, and the levels between them, the *Microcystis* dominance could shift to diatom dominance, particularly *Nitzschia palea* dominance. During the algal shift under the aeration mixing, the colonial *Microcystis* decayed and released soluble inorganic nitrogen and phosphorus; however, enrichment of more soluble inorganic nitrogen and phosphorus was beneficial for the *Nitzschia* increase in the high biomass treatment alone. The *Nitzschia* cells were in aggregates with the *Microcystis* colonies instead of free-living, and the mucilaginous sheath of the *Microcystis* colonies provided the physical medium for the aggregation. This study found for the first time that *Microcystis* blooms could shift to *Nitzschia* dominance in aggregates. This experiment provided a method to control and manipulate *Microcystis* blooms to diatom dominance through continuous aeration mixing to proper biomass of *Microcystis* colonies. The shift to diatoms dominance would provide more high quality food organisms for aquaculture and be beneficial to the material cycling and energy flowing in food web dynamics.

## Data Availability

All data generated or analyzed during this study are included in this article. If required, data will be provided separately upon request.

## References

[1] Xu H, Paerl HW, Qin B, Zhu G, Gao G (2010). Nitrogen and phosphorus inputs control phytoplankton growth in eutrophic Lake Taihu. China. Limnol. Oceanogr..

[2] Elliott JA (2012). Is the future blue-green? A review of the current model predictions of how climate change could affect pelagic freshwater cyanobacteria. Water Res..

[3] Paerl HW, Otten TG (2013). Harmful cyanobacterial blooms: causes, consequences, and controls. Microb. Ecol..

[4] Huisman J, Codd GA, Paerl HW, Ibelings BW, Verspagen JM, Visser PM (2018). Cyanobacterial blooms. Nat. Rev. Microbiol..

[5] Ho JC, Michalak AM, Pahlevan N (2019). Widespread global increase in intense lake phytoplankton blooms since the 1980s. Nature.

[6] Carmichael WW (2001). Human fatalities from cyanobacteria: chemical and biological evidence for cyanotoxins. Environ. Health. Persp..

[7] Lyu K (2016). Changes in iTRAQ-based proteomic profiling of the cladoceran *Daphnia magna* exposed to microcystin-producing and microcystin-free *Microcystis aeruginosa*. Environ. Sci. Technol..

[8] Shahmohamadloo RS (2021). Cyanotoxins within and outside of *Microcystis aeruginosa* cause adverse effects in rainbow trout (*Oncorhynchus mykiss*). Environ. Sci. Technol..

[9] Malbrouck C, Kestemont P (2006). Effects of microcystins on fish. Environ. Toxicol. Chem..

[10] Shahmohamadloo RS, Bhavsar SP, Almirall XO, Marklevitz SA, Rudman SM, Sibley PK (2023). Lake Erie fish safe to eat yet afflicted by algal hepatotoxins. Sci. Total Environ..

[11] Stroom JM, Kardinaal WEA (2016). How to combat cyanobacterial blooms: strategy toward preventive lake restoration and reactive control measures. Aquat. Ecol..

[12] Lürling M, Mucci M (2020). Mitigating eutrophication nuisance: in-lake measures are becoming inevitable in eutrophic waters in the Netherlands. Hydrobiologia.

[13] Wang Z (2021). Cyanobacterial dominance and succession: Factors, mechanisms, predictions, and managements. J. Environ. Manag..

[14] Zhu K, Bi Y, Hu Z (2013). Responses of phytoplankton functional groups to the hydrologic regime in the Daning River, a tributary of Three Gorges Reservoir. China. Sci. Total Environ..

[15] Qu Y, Wu N, Guse B, Fohrer N (2018). Riverine phytoplankton shifting along a lentic-lotic continuum under hydrological, physiochemical conditions and species dispersal. Sci. Total Environ..

[16] Visser PM, Ibelings BW, Van Der Veer B, Koedood J, Mur R (1996). Artificial mixing prevents nuisance blooms of the cyanobacterium *Microcystis*, in Lake Nieuwe Meer, the Netherlands. Freshw. Biol..

[17] Jungo E, Visser PM, Stroom J, Mur LR (2001). Artificial mixing to reduce growth of the blue-green alga *Microcystis* in Lake Nieuwe Meer, Amsterdam: an evaluation of 7 years of experience. Water Sci. Tech-W. Sup..

[18] Huisman J (2004). Changes in turbulent mixing shift competition for light between phytoplankton species. Ecology.

[19] Ferris JA, Lehman JT (2007). Interannual variation in diatom bloom dynamics: Roles of hydrology, nutrient limitation, sinking, and whole lake manipulation. Water Res..

[20] Zhou J (2015). Effects of wind wave turbulence on the phytoplankton community composition in large, shallow Lake Taihu. Environ. Sci. Pollut. Res..

[21] Hambright KD, Zohary T (2000). Phytoplankton species diversity control through competitive exclusion and physical disturbances. Limnol. Oceanogr..

[22] Visser PM, Ibelings BW, Bormans M, Huisman J (2016). Artificial mixing to control cyanobacterial blooms: A review. Aquat. Ecol..

[23] Wilkinson A, Hondzo M, Guala M (2016). Effect of small-scale turbulence on the growth and metabolism of *Microcystis aeruginosa*. Adv. Microbiol..

[24] Zhu M (2014). The role of tropical cyclones in stimulating cyanobacterial (*Microcystis* spp.) blooms in hypertrophic Lake Taihu, China. Harmful Algae.

[25] Yang G (2020). Intermittent disturbance benefits colony size, biomass and dominance of *Microcystis* in Lake Taihu under field simulation condition. Harmful Algae.

[26] Yang G (2022). Continuous hydrodynamic mixing weakens the dominance of *Microcystis*: evidences from microcosm and lab experiments. Environ. Sci. Pollut. Res..

[27] Wang X (2022). The biomass of bloom-forming colonial *Microcystis* affects its response to aeration disturbance. Sci. Rep..

[28] Jensen JR, Jeppesen E, Olrik K, Kristensen R (1994). Impact of nutrients and physical factors on the shift from Cyanobacteria to Chlorophyte in shallow Danish lakes. Can. J. Fish. Aquat. Sci..

[29] Chen Y, Qin B, Teubner K, Dokulil MT (2003). Long-term dynamics of phytoplankton assemblages: *Microcystis-*domination in Lake Taihu, a large shallow lake in China. J. Plankton Res..

[30] Ma J (2015). Green algal over cyanobacterial dominance promoted with nitrogen and phosphorus additions in a mesocosm study at Lake Taihu. China. Environ. Sci. Pollut. Res..

[31] Yang R, Wei D, Xie J (2020). Diatoms as cell factories for high-value products: chrysolaminarin, eicosapentaenoic acid, and fucoxanthin. Crit. Rev. Biotechnol..

[32] Viana MT, Correa G, Lazo JP, Frías-Díaz R, Durazo-Beltrán E, Vasquez-Pelaez C (2007). Digestive physiology and metabolism of green abalone *Haliotis fulgens* from postlarvae to juvenile, fed three different diatoms. Aquaculture.

[33] Gross A, Boyd CE (1998). A digestion procedure for the simultaneous determination of total nitrogen and total phosphorus in pond water. J. World Aquacult. Soc..

[34] Eaton AD, Clesceri LS, Greenburg AE (1995). Standard methods for examination of water and wastewater.

[35] Palmer CM, Maloney TE (1954). A new counting slide for nanoplankton. Limnol. Oceanogr..

[36] Hu H, Wei Y (2006). The freshwater algae of China—systematics, taxonomy and ecology.

[37] Underwood AJ (1997). Experiments in ecology: Their logical design and interpretation using analysis of variance.

[38] Moreno-Ostos E, Cruz-Pizarro L, Basanta A, George DG (2009). The influence of wind-induced mixing on the vertical distribution of buoyant and sinking phytoplankton species. Aquat. Ecol..

[39] Reynolds CS, Wiseman SW, Godfrey BM, Butterwick C (1983). Some effects of artificial mixing on the dynamics of phytoplankton populations in large limnetic enclosures. J. Plankton Res..

[40] Hawkins PR, Griffiths DJ (1993). Artificial destratification of a small tropical reservoir: effects upon the phytoplankton. Hydrobiologia.

[41] Kang L (2019). Interactions between suspended particulate matter and algal cells contributed to the reconstruction of phytoplankton communities in turbulent waters. Water Res..

[42] Neale PJ, Heaney SI, Jaworski GHM (1991). Responses to high irradiance contribute to the decline of the spring diatom maximum. Limnol. Oceanogr..

[43] Huisman J, Oostveen PV, Weissing FJ (1999). Species dynamics in phytoplankton blooms: incomplete mixing and competition for light. Am. Nat..

[44] Thomas WH, Gibson CH (1990). Effects of small-scale turbulence on microalgae. J. Appl. Phycol..

[45] Xu H, Jiang H, Yu G, Yang L (2014). Towards understanding the role of extracellular polymeric substances in cyanobacterial *Microcystis* aggregation and mucilaginous bloom formation. Chemosphere.

[46] Le VV, Srivastava A, Ko SR, Ahn CY, Oh HM (2022). *Microcystis* colony formation: Extracellular polymeric substance, associated microorganisms, and its application. Bioresour. Technol..

[47] Shao K (2014). The responses of the taxa composition of particle-attached bacterial community to the decomposition of Microcystis blooms. Sci. Total Environ..

[48] Zohary T, Pais-Madeira AM, Robarts RD, Hambright KD (1996). Interannual phytoplankton dynamics of hypertrophic African lake. Arch. Hydrobiol..

[49] Thornton DCO (2002). Diatom aggregation in the sea: Mechanisms and ecological implications. Eur. J. Phycol..

[50] Thornton DCO, Chen J (2017). Exopolymer production as a function of cell permeability and death in a diatom (*Thalassiosira weissflogii*) and a cyanobacterium (*Synechococcus elongatus*). J. Phycol..

[51] Sazhin AF, Artigas LF, Nejstgaard JC, Frischer ME (2007). The colonization of two *Phaeocystis* species (*Prymnesiophyceae*) by pennate diatoms and other protists: A significant contribution to colony biomass. Biogeochemistry.

[52] Rousseau V (1994). The life cycle of *Phaeocystis* (Prymnesiophycaea): Evidence and hypotheses. J. Marine. Syst..

[53] Hamm CE, Rousseau V (2003). Composition, assimilation and degradation of *Phaeocystis globosa*-derived fatty acids in the North Sea. J. Sea. Res..

